# Ulinastatin: A Potential Alternative to Glucocorticoid in the Treatment of Severe Decompression Sickness

**DOI:** 10.3389/fphys.2020.00273

**Published:** 2020-03-26

**Authors:** Wen-tao Meng, Long Qing, Chun-zhen Li, Kun Zhang, Hong-jie Yi, Xu-peng Zhao, Wei-gang Xu

**Affiliations:** ^1^Department of Diving and Hyperbaric Medicine, Naval Special Medicine Center, Naval Medical University, Shanghai, China; ^2^Naval Diving Medical Discipline, Naval Special Medicine Center, Naval Medical University, Shanghai, China; ^3^School of Basic Medicines, Naval Medical University, Shanghai, China

**Keywords:** ulinastatin, decompression sickness, inflammation, endothelial injury, rabbit

## Abstract

Inflammatory reaction is the crux in various clinical critical diseases including decompression sickness (DCS). Ulinastatin (UTI), a potent anti-inflammatory agent, has been used clinically, including as a substitution for steroids. This study aimed to explore the potential effects of UTI upon DCS in a rabbit model. Eighty-eight rabbits were subjected to simulated diving to 6 atmospheres absolute (ATA) for 60 min with 2.5-minute decompression. Three doses of UTI (15/7.5/3.75 × 10^5^ U/kg) or saline were intravenously administered immediately following decompression. Circulating bubbles were monitored for 3 h following decompression and DCS signs were evaluated for 24 h. Blood was sampled 8 times during 72 h after decompression for inflammatory, endothelial, oxidative and routine blood indices. Lung tissues were also sampled for evaluating endothelial function. Another six rabbits were used as Normal controls. In the high dose UTI group the mortality, general morbidity and incidence of severe DCS was decreased from 31.25 to 9.38% (*P* = 0.030), 84.38 to 62.50% (*P* = 0.048) and 46.88 to 21.88% (*P* = 0.035), respectively. The high dose of UTI significantly postponed the occurrence of DCS (*P* = 0.030) and prolonged survival time (*P* = 0.009) compared with the Saline group, and significantly ameliorated inflammation responses, endothelial injuries and oxidative damage. The results strongly suggest the benefit of UTI on DCS, especially for severe cases. Large doses are needed to achieve significant effects. UTI may be a potential ideal pharmacological candidate for the treatment of severe DCS.

## Introduction

Urinary trypsin inhibitor (Ulinastatin, UTI) is a nonspecific and multivalent Kunitz-type serine protease inhibitor purified from human urine (Yano et al., 2003). It is capable of suppressing various serine proteases such as trypsin, plasmin, neutrophil elastase and chymotrypsin (Nishiyama et al., 1996; Nakatani et al., 2001), and can also effectively stabilize lysosomal and cellular membranes (Nakahama et al., 1999). UTI has confirmed powerful efficacy in inhibiting the release of inflammatory factors, removing oxygen free radicals, improving microcirculation and tissue perfusion, and alleviating endothelial injuries (Takada et al., 2003; Tanaka et al., 2010; We et al., 2017; Fang et al., 2018). Clinically, UTI has been applied effectively in treating acute pancreatitis, acute circulatory failure, and ischemia-reperfusion injury with few adverse events (Yoo et al., 2008; Li et al., 2017). Recently, UTI was demonstrated to exert protective effects in shock (Li et al., 2019). These clinical indications are similar to those of many steroids.

Decompression sickness (DCS) is one major concern for divers, astronauts and other personnel engaging in barometric operations, and bubble formation in tissue and circulation is the underlying mechanism (Arieli, 2018). As foreign bodies, bubbles can activate inflammatory cascade reactions, and induce cell irritation and tissue injuries (Papadopoulo et al., 2014). In the severe forms of DCS, significant neurological, respiratory and circulatory deficits or failure are frequently involved, and steroids are routinely used due to their potency on inhibiting systemic inflammation, reducing capillary permeability, maintaining cell membrane and reducing tissue edema (Kizer, 1981). However, many studies have shown no significant improvement of DCS when treated with steroids (Dromsk et al., 2003; Montcalm-Smith et al., 2008), and may even worsen the outcome of central nervous system injury due to their adverse effect of elevating blood glucose (Chikani et al., 2017). Hence, there has been a decline in the use of steroids for the treatment of severe DCS as well as other critical diseases due to their adverse effects and uncertainty of outcomes (Dromsk et al., 2003; Felleiter et al., 2012; Gibbison et al., 2017; Walje et al., 2017; Oh et al., 2019). Since 2008, the United States Navy no longer recommends steroids for DCS treatment. In consideration of UTI’s unique pharmacological properties, we speculated that UTI would have therapeutic effects on DCS, and the underlying mechanisms are also explored in the present study.

## Materials and Methods

### Animals

Ninety-four male *New Zealand White* rabbits weighing 2.0∼2.3 kg were obtained from Shanghai Shengwang Laboratory Animal Co., Ltd. The current study was performed at the laboratory of Diving and Hyperbaric Medicine at the Naval Medical University. All experimental procedures in this study were reviewed and approved by the Ethics Committee for Animal Experiment of the university. The rabbits were housed, and standard chow and water were given *ad libitum* in a room with controlled humidity (50∼60%), temperature (24∼26°C) and a 12-hour light-dark cycle.

### Experimental Procedure and Design

After acclimation to the laboratory environment for one week, 88 rabbits were randomly divided into 4 groups and subjected to simulated diving and rapid decompression. After surfacing, either a single intravenous injection of UTI (Techpool Biopharma Co., Ltd., Guangdong, China) at 15 × 10^5^ U/kg (High dose, UTI-H, *n* = 32), 7.5 × 10^5^ U/kg (Median dose, UTI-M, *n* = 12) and 3.75 × 10^5^ U/kg (Low dose, UTI-L, *n* = 12) or a same volume of saline (*n* = 32) were immediately administered. UTI was dissolved in saline (1 ml/kg body weight) and injected via the marginal ear veins. All of the animals were under continuous observation for 24 h after decompression to evaluate DCS signs. Blood was sampled before the simulated diving and at 1, 6, 12, 24, 36, 48, and 72 h following decompression to determine serum levels of biochemical indices. Twelve rabbits in each group underwent bubble detection at 10, 20, 30, 40, 60, 90, 120, and 180 min following decompression, and were then euthanized by intraperitoneal injection of pentobarbital (200 mg/kg) for sampling lung tissues and bronchoalveolar lavage fluid (BALF). Another six rabbits were sham exposed at normobaric air in the chamber as normal controls and were similarly sampled.

### Hyperbaric Exposure Protocol

The rabbits were subjected to simulated diving in pairs as designated in Table 1 in an animal hyperbaric chamber (DWC150, Yangyuan, Shanghai, China). The pressure was increased to six atmospheres absolute (ATA) in 5 min, slowly at the beginning to minimize any possible discomfort, and maintained for 60 min before decompressing linearly at two ATA/min to ambient pressure. Our previous research showed this profile could produce an incidence of DCS in rabbits of around 75% (Meng et al., 2018). During exposure, the temperature inside the chamber was maintained between 23 and 25°C, and the chamber was ventilated continuously for timely removal of metabolic generated carbon dioxide (CO_2_).

**TABLE 1:** The matching scheme of rabbits from different groups for simulated diving.

Group	UTI-H	UTI-M	UTI-L	Saline	Sum
UTI-H	–	4	4	24	32
UTI-M	4	–	4	4	12
UTI-L	4	4	–	4	12
Saline	24	4	4	–	32

### DCS Symptoms Observation and Functional Evaluation

Following decompression, DCS was evaluated by two observers who were blinded to the treatments. The appearance and onset time of DCS signs were recorded. Severe DCS was defined as individuals with at least one of the signs including paralysis, dyspnea, seizure and death. Respiratory function was monitored and scored at 10, 20, 30, 40, 60, 90, and 120 min post decompression using a 0–4 grading system as following (Atkins et al., 1988): 0, normal breathing; 1, mild labored breathing; 2, restlessness and labored breathing; 3, severely labored breathing, recumbent posture; 4, collapse, stupor and death. Motor function was evaluated before simulated diving and at 1, 6, 12, and 24 h after surfacing using Tarlov score (Kertmen et al., 2013), and each rabbit was scored 0 to 5 as following: 0, normal hind-limb function; 1, able to hop but uncoordinated; 2, able to sit but unable to hop; 3, active movement but unable to sit without assistance; 4, movement of joints perceptible; and 5, no voluntary hind-limb movement.

### Bubble Detection

After surfacing, rabbits were placed in a supine position for bubble detection using a 10 MHz transducer connected to an ultrasound scanner (Mylab 30CV, Esaote, Italy). Bubbles were recognized as bright spots in heart chambers, and were scored by optimized Eftedal-Brubakk (EB) grading scale (Møllerløkken et al., 2016), with the modification shown in Table 2.

**TABLE 2:** Modified Eftedal-Brubakk score for ultrasound-detected bubbles.

Score	Definition
0	No visible bubbles
1	Occasional bubbles
2	At least 1 bubble every 4 heart cycles
3	At least 1 bubble every heart cycle
4	Not more than one third of every image
5	Not more than two thirds of every image
6	Near whiteout; individual bubbles still discerned
7	Whiteout; individual bubbles can’t be discerned

### Blood Routine Examination

Half milliliter venous blood samples were taken from the marginal ear veins and collected into EDTA-anticoagulated tubes for blood routine examination using an automatic hematology analyzer (BC-2800Vet, Mindray, China).

### Determination of Serum Biochemical Indices

Another 2 ml venous blood was obtained and centrifuged at 4°C and 2500 rpm for 10 min. Serum levels of intercellular cell adhesion molecule-1 (ICAM-1), endothelin-1 (ET-1), monocyte chemoattractant protein-1 (MCP-1) and interleukin-1beta (IL-1β) were determined by respective enzyme-linked immunosorbent assay (ELISA) kits (Jiancheng Bioengineering Institute, Nanjing, China). Levels of malondialdehyde (MDA) and myeloperoxidase (MPO) were determined by ELISA using respective assay kits (Melian Biological Technology Co., Shanghai, China). All assays were performed according to the manufacturers’ instructions.

### Lung Wet/Dry Weight Ratio Assay and BALF Analysis

After euthanasia at 24 h following decompression, fresh specimens of the right lung lobes were removed and weighed as wet weight and placed in an oven at 60°C for 72 h to measure dry weight. Lung water content was assayed by Wet/Dry (W/D) weight ratio. A plastic tube was tied into the trachea and the left lung lavage was performed by flushing the airways with 3 × 10 ml 0.9% saline, and BALF recovery was approximately 80%. Total BALF protein was measured by bicinchoninic acid (BCA) using enhanced BCA protein assay kit (Beyotime Institute of Biotechology, Nantong, China).

### Statistical Analysis

Where applicable, parametric data are expressed as mean ± s.d. Morbidity and mortality from different treatments were compared by Chi-square test. Normal distribution was determined by Shapiro-Wilk test. Comparisons of latency to DCS and survival curves were performed with the log-rank test. Bubble loads were analyzed by the generalized estimation equation. The mean differences between groups were assessed using an LSD test or Dunnett’s test. Respiratory and Tarlov scores were compared between UTI and Saline groups by Mann-Whitney *U* test. *P* values less than 0.05 were considered statistically significant.

## Results

### Morbidity and Mortality of DCS

**FIGURE 1:**
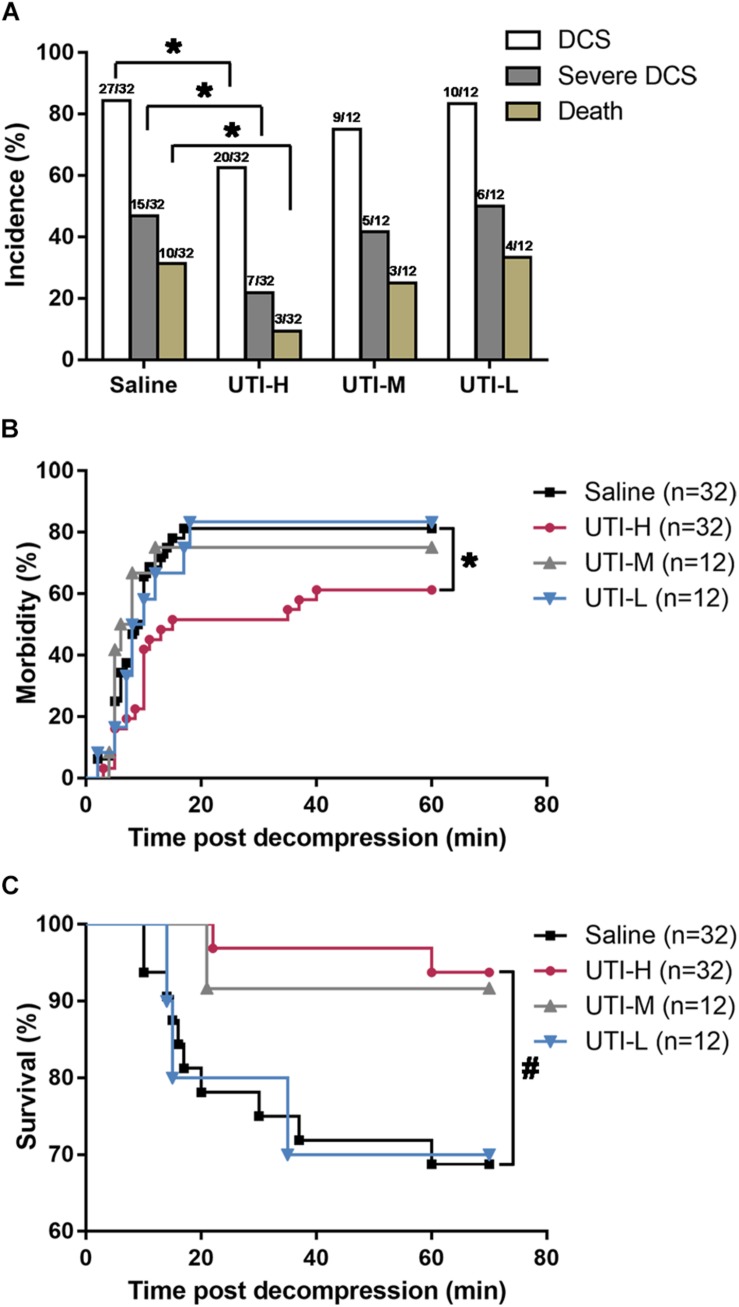
Effects of different doses of UTI on DCS morbidity and mortality in rabbits. Rabbits were subjected to a simulated dive to six atmospheres absolute (ATA) for 60 min with 2.5-minute decompression, and received intravenous injection of different doses of UTI or saline immediately following decompression. **(A)** The mortality, general morbidity and incidence of severe DCS. **(B)** The incidence of DCS with time. **(C)** Survival curve of rapid decompressed rabbits. * *P* < 0.05, ^#^*P* < 0.01 vs. Saline group.

The rabbits weighted 2.16 ± 0.12 kg before simulated diving, and there was no significant difference between groups. In 95% animals, DCS occurred within 30 min following decompression. The morbidity of DCS and severe DCS in the UTI-H group were significantly lower than in the Saline group (62.50% vs. 84.38%, χ^2^ = 3.925, *P* = 0.048 and 21.88% vs. 46.88%, χ^2^ = 4.433, *P* = 0.035; Figure 1A), and the occurrence of DCS was significantly postponed (χ^2^ = 4.724, *P* = 0.030, Figure 1B). The majority of deaths occurred following ∼1 min severe dyspnea and a transient convulsion. The high dose of UTI significantly decreased mortality from 31.25 to 9.38% (χ^2^ = 4.730, *P* = 0.030, Figure 1A), and survival time was also obviously extended (χ^2^ = 6.838, *P* = 0.009, Figure 1C). No statistical significance was found in DCS signs between UTI-M, UTI-L and Saline groups.

### Bubble Load Following Decompression

**FIGURE 2:**
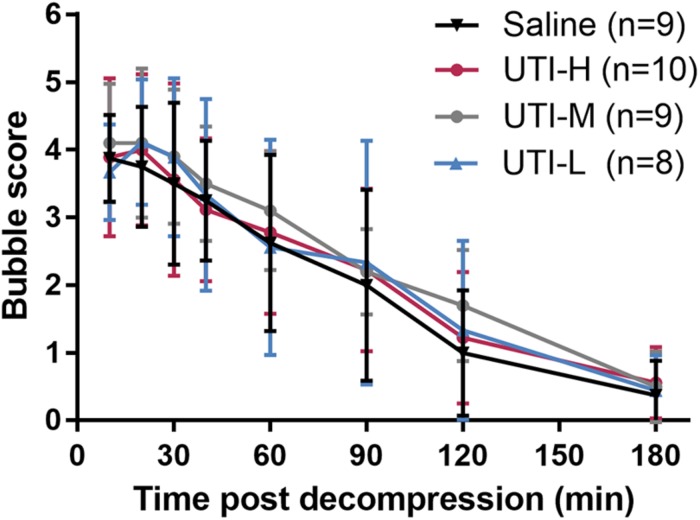
Bubble load in rabbits after simulated diving and UTI treatments. After a simulated dive, rabbits immediately received an intravenous injection of different doses of UTI or saline. Bubbles were detected by ultrasound at multiple time points following decompression and were scored using the modified Eftedal-Brubakk grading scale. No statistical difference existed between groups (*P* = 0.895).

Abundant bubbles were detected as bright spots in the right heart chambers. Bubbles peaked within 30 min following decompression, and diminished gradually thereafter (Figure 2). There was no obvious difference between bubble scores in rabbits treated with UTI and saline (*F* = 0.201 and *P* = 0.895).

### Protective Effects of UTI Against Lung Injuries and Neurological Disorders

**FIGURE 3:**
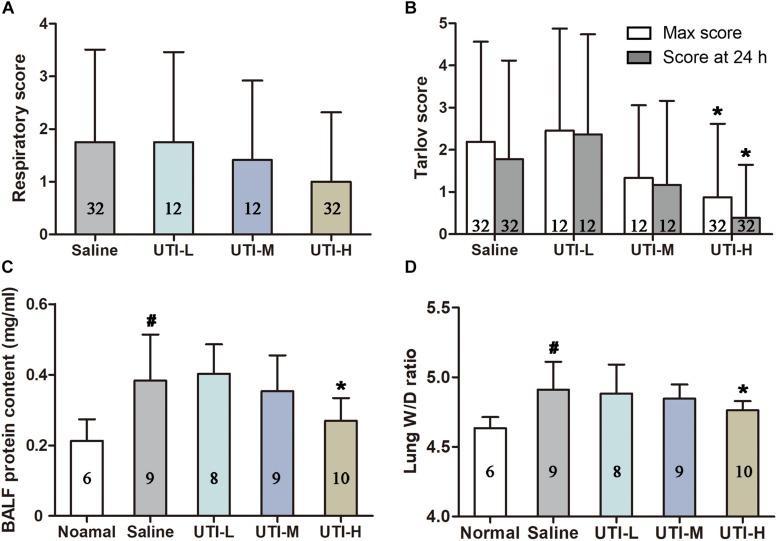
Protective effects of UTI against lung injuries and neurological disorders. Respiratory function was monitored and scored for 2 h following simulated diving. The maximum observed respiratory scores are shown in panel **(A)**. Motor function was evaluated using Tarlov score for 24 h, and UTI-H significantly lowered both the maximal observed scores and scores assessed at 24 h after surfacing **(B)**. The high dose of UTI reduced the elevated BALF protein content and lung W/D ratio **(C,D)**. *^#^P* < 0.01 vs. Normal controls, * *P* < 0.05 vs. Saline group.

In the Saline group, 37.50% of individuals underwent a short period of fast or difficult breathing following decompression. Though no obvious difference existed in UTI treated animals when compared with those treated with saline, the high dose of UTI showed a tendency to decrease maximum respiratory scores observed during the 2 h following decompression (*Z* = −1.684, *P* = 0.092, Figure 3A). Protein content in BALF and lung W/D weight ratio were significantly decreased in the UTI-H group compared with the Saline group (*P* = 0.025, Figure 3C and *P* = 0.042, Figure 3D), and detailed statistical results are shown in Table 3. There was no statistical difference in UTI-M and UTI-L with regard to BALF protein (*P* = 0.592 and *P* = 0.727, Figure 3C) or lung W/D weight ratio (*P* = 0.402 and *P* = 0.781, Figure 3D). 46.88% of individuals in the Saline group developed neurological disorders, which were accompanied with different degrees of paralysis. The high dose of UTI significantly lowered maximal observed Tarlov scores and scores assessed at 24 h following decompression (*Z* = −2.220, *P* = 0.026, and Z = −2.592, *P* = 0.010, Figure 3B). The median or low doses of UTI did not show a statistical difference.

**TABLE 3:** Results of ANOVA and multiple comparisons.

Parameters	Testing time (time post de- compression)	ANOVA			*Post Hoc* Tests	
			
		*df*	*F*	*P*	Groups (vs. Saline group)	*P*
W/D weight ratio	24 h	4	4.120	0.007	UTI-H	0.034
					UTI-M	0.352
					UTI-L	0.693
					Normal	0.001
BALF Protein	24 h	4	5.492	0.001	UTI-H	0.012
					UTI-M	0.498
					UTI-L	0.675
					Normal	0.001
WBC	1 h	3	2.928	0.049	UTI-H	0.006
					UTI-M	0.066
					UTI-L	0.134
PLT	1 h	3	3.047	0.043	UTI-H	0.005
					UTI-M	0.065
					UTI-L	0.160
IL-1β	6 h	3	7.195	0.000	UTI-H	0.001
					UTI-M	0.018
					UTI-L	0.999
MCP-1	12 h	3	6.897	0.000	UTI-H	0.002
					UTI-M	0.106
					UTI-L	0.181
ET-1	12 h	3	9.554	0.000	UTI-H	0.001
					UTI-M	0.011
					UTI-L	0.340
ICAM-1	12 h	3	13.733	0.000	UTI-H	0.000
					UTI-M	0.000
					UTI-L	0.022
MDA	6 h	3	5.560	0.002	UTI-H	0.001
					UTI-M	0.174
					UTI-L	0.806
MPO	1 h	3	2.799	0.047	UTI-H	0.006
					UTI-M	0.083
					UTI-L	0.273

*Rabbits were subjected to simulated diving to 6 atmospheres absolute (ATA) for 60 min with 2.5-minute decompression, and received intravenous injection of different doses of UTI or saline immediately following decompression. Lung tissues and BALF were sampled for determination of W/D weight ratio and protein content, respectively. Blood was sampled for WBC, PLT and serum indices including IL-1β, MCP-1, ET-1, ICAM-1, MDA, and MPO. Values of the indices were compared between the Saline group and UTI groups at different time points.*

### Protective Effects of UTI on Blood Routine

**FIGURE 4:**
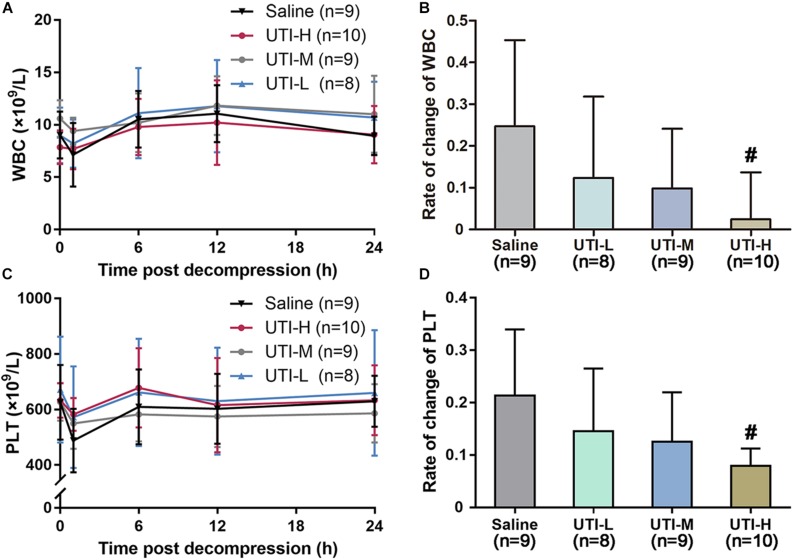
Protective effects of different doses of UTI on blood routine. Blood was sampled from marginal ear veins in rabbits treated with saline or UTI before and after a simulated dive. Changes of WBC and PLT with time are shown **(A,C)**, and respective changes rates of different groups at 1 h following decompression are shown in panels **(B,D)**. The change rates of WBC and PLT were compared between UTI and Saline groups, respectively. *^#^P* < 0.01 vs. Saline group.

As shown in Figure 4, counts of white blood cells (WBC) and platelet (PLT) decreased rapidly 1 h following decompression, and returned to normal gradually around 24 h following decompression. The high dose of UTI significantly inhibited the reduction of WBC and PLT (*P* = 0.006, Figure 4B and *P* = 0.005, Figure 4D). No statistical difference existed in UTI-M and UTI-L in terms of WBC (*P* = 0.066 and *P* = 0.134, Figure 4B) and PLT (*P* = 0.065 and *P* = 0.160, Figure 4D).

### Anti-inflammatory, Anti-oxidative, Endothelial Protective Effects of UTI Against DCS

**FIGURE 5:**
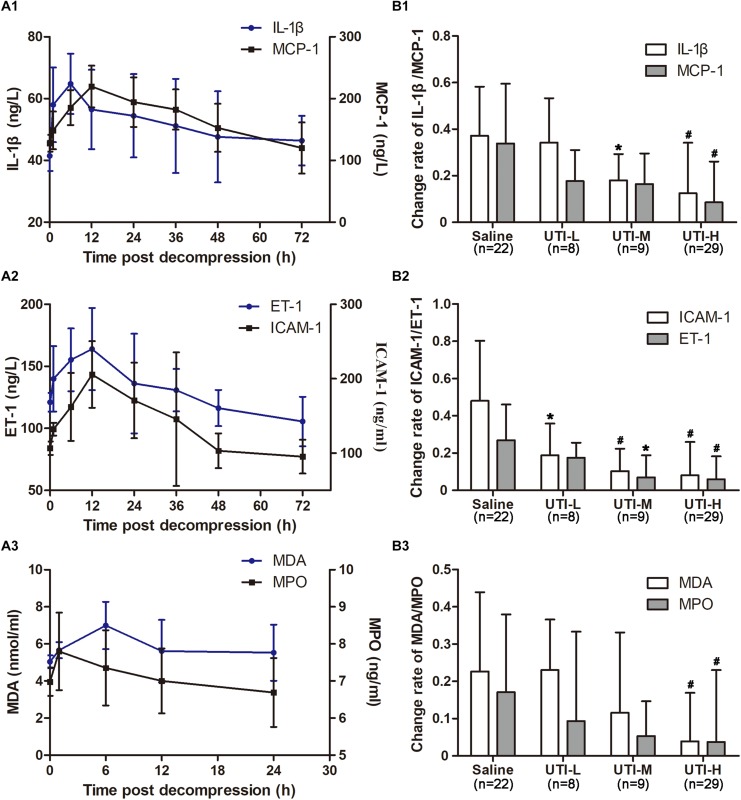
Effects of different doses of UTI on inflammatory, endothelial and oxidative indicators. Before simulated diving and 1, 6, 12, 24, 36, 48, and 72 h following decompression, blood was sampled via marginal ear veins in rabbits treated with different doses of UTI or saline. Six rabbits in the Saline group were used to monitor changes in biochemical indices over time to determine their respective peak time points **(A1–A3)**, and the change rates were compared between UTI and Saline groups at the respective peak time points **(B1–B3)**. *^∗^P* < 0.05 and *^#^P* < 0.01 vs. Saline group.

Levels of IL-1β, MCP-1, ET-1, ICAM-1, MDA, and MPO increased following decompression, peaked at 6, 12, 12, 12, 6, and 1 h, and gradually decreased to normal around 72, 72, 48, 48, 24, and 24 h, respectively (Figures 5A1–A3). As shown in Figures 5B1–B3, both the high and median doses of UTI reduced the peak levels of all the determined biochemical indices (*P* < 0.05 or *P* < 0.01) except median dose of UTI on MCP-1, MDA and MPO, which did not reach statistical significance. The low dose of UTI decreased MCP-1, ET-1, ICAM-1 and MPO levels, but only reached statistical significance on ICAM-1 (*P* < 0.05). Statistical results in detail are shown in Table 3.

## Discussion

Inflammation is an adaptive and evolving process, which can be triggered by pernicious stimuli and conditions, such as infection and tissue injury (Medzhitov, 2008). Generally, it is supposed to be beneficial when it’s under control, such as by exerting protective effects against infection. However, it becomes detrimental when out of control and may progress into a vicious cycle. Decompression bubbles formed in tissues and the circulatory system trigger inflammatory cascades, and play an extremely important role in the pathophysiology of DCS, especially in severe cases, in which systemic inflammatory responses continue despite the disappearance of bubbles, and further worsen the outcomes of DCS (Levin et al., 1981; Dennison et al., 2012). The inhibition of inflammation cascade responses is of great importance to stop the progression of DCS (Papadopoulo et al., 2014).

Ulinastatin is a multivalent serine protease inhibitor extracted from human urine. It exerts protective effects via suppressing various serine proteases (Nishiyama et al., 1996; Park et al., 2010) and stabilizing lysosomes and cellular membranes (Kanayama et al., 1997). Various clinical and animal experiments have demonstrated its anti-inflammatory, anti-oxidative, anticoagulant properties, and it can also improve the microcirculatory environment and reduce tissue damage (Tsujimura et al., 2008; Xu et al., 2012; Li et al., 2017; Liu et al., 2018). Clinically, it has been adopted successfully in the treatment of life-threatening inflammation such as acute pancreatitis and sepsis (Tsujino et al., 2005; Zhang et al., 2008). Previously, steroids were routinely recommended and administered for severe DCS considering their benefits of decreasing tissue edema, reducing inflammation, alleviating leakage of blood vessels, and preventing histamine release (Kizer, 1981; Halpern et al., 1982; Dutka et al., 1992; Montcalm-Smith et al., 2008). However, even a high dose of steroids showed no improvement (Francis and Dutka, 1989; Montcalm-Smith et al., 2008) and even worsened the outcomes of DCS in animals (Dromsk et al., 2003). Side effects including the elevation of blood sugar were observed and steroids are no longer recommended for treating DCS in divers (Moon, 2009). Since UTI is regarded as an exciting alternative for steroids clinically (Linder and Russell, 2014), research into the potential protective effects of UTI on DCS is warranted.

In this study, the median dose of 7.5 × 10^5^ U/kg was derived from the effective dosage commonly used in experimental acute pancreatitis (Ohbishi et al., 1984), and a dose gradient of 3.75/7.5/15 × 10^5^ U/kg was adopted to explore possible dose-effect responses. Based on our preliminary experiments, the morbidity of DCS was around 75% for the saline treated rabbits and 40% for high dose of UTI treated ones, and a sample size of 32 for the two groups would provide 80% power to reach statistical significance concerning DCS morbidity based on a two-tailed significance level of 0.05. To minimize the use of animals, 12 rabbits were chosen for the other two dosage groups, which were designed to compare mainly biochemical indices with a concurrent observation of morbidity and mortality.

Autochthonous or circulating bubbles formed after rapid decompression play an important role in the progress of DCS (Qing et al., 2017; Zhang et al., 2017). The bubbles can not only cause mechanical damages and blockage, but also act as foreign bodies that trigger a cascade of inflammatory responses (Levett and Millar, 2008). As a powerful serine protease inhibitor, UTI is capable of reducing the release of inflammatory factors (Liu et al., 2017). IL-1β is one proximal factor responsible for downstream cytokine production and vascular injuries induced by decompression (Thom et al., 2018). MCP-1 is another proinflammatory cytokine in the process of disease resistance, which is related to decompression stress (Qing et al., 2018). UTI treatment significantly decreased the elevated serum levels of IL-1β and MCP-1 and, hence, in this study relieved inflammation cascade reactions induced by DCS.

In addition to its anti-inflammation property, UTI also effectively relieves endothelial injuries in DCS, probably through protecting endothelial glycocalyx (Wang et al., 2017). Besides, UTI can reduce the permeability of pulmonary capillary endothelial cells through the protection of endothelial junctional proteins (Fang et al., 2018). UTI may also protect endothelial cells from neutrophil-mediated injury not only by inactivation of the extracellular elastase excreted from neutrophils, but also by suppressing the production of activated elastase (Nakatani et al., 2001). These pathways ultimately lowered the degrees of endothelial injuries and high permeability. Since the endothelial system is one of the vulnerable organs of DCS, UTI may hence alleviate DCS injuries. The effectiveness on reducing BALF protein content and lung W/D weight ratio, and serum levels of ET-1 and ICAM-1 provide evidence that endothelial injuries and microvascular permeability were ameliorated by UTI.

Bubbles interact with blood cells and plasma proteins, which may cause platelet activation and deposition, as well as leucocyte-endothelial adhesion, which are further enhanced by the increased expression of ICAM-1 (Aosasa et al., 1998; Levett and Millar, 2008; Vann et al., 2011). UTI has been confirmed to be effective in inhibiting neutrophil accumulation caused by the ischemia/reperfusion injury (Okuhama et al., 1999), and suppressing reduction of PLT induced by lipopolysaccharide (Inoue and Takano, 2010). Similarly, in this study UTI effectively counteracted the reduction of WBC and PLT post decompression. Apart from the properties mentioned above, UTI has also been demonstrated to inhibit oxidative stress and acts as a potent reactive oxygen scavenger (Du and Ko, 2005). Levels of oxidative stress, which were correlated with the severity of decompression (Lambrechts et al., 2015), were significantly lowered in UTI-H as exhibited by the changes of MDA and MPO. As oxidative stress is likely to trigger endothelial responses (Eftedal et al., 2012), endothelial injuries may be relieved in turn due to alleviated oxidative stress.

The inflammatory, endothelial and oxidative indices in this study were mainly selected based on our previous studies in rat and swine DCS models (Zhang et al., 2017; Qing et al., 2018, 2019). In accordance with our swine model, all biochemical indices showed similar changes to those seen in rats except for E-selectin, which showed no change in the rabbit and swine DCS model. Considering the time course following decompression of these indices between different species, there was a general trend showing that the peak time points in rabbits were earlier than that of swine, then that of rats. These time courses supply indispensable information to determine the severity of DCS at different times following decompression. Another trend observed was that the change rates of indices were contrary to animal size.

The present results also confirm the protective effects of UTI against lung injuries and neurological disorders induced by DCS. There is strong evidence indicating protective effects of UTI against sepsis-induced multiorgan injuries, such as acute lung injury (Fang et al., 2018). It has been demonstrated that UTI relieves TNF-α induced hyperpermeability of vascular endothelial cells (We et al., 2017). In addition, UTI can effectively inhibit inflammation and reduce protein expression of AQP-4 (Cui and Zhu, 2015), which helps to attenuate capillary permeability and, as a result, alleviates lung and neural injuries.

The results of the present study show that a high dose of UTI (15 × 10^5^ U/kg) significantly lowered the morbidity and mortality of DCS. Because no obvious difference in circulating bubbles between groups were found, the protective effects of UTI relied mainly on its anti-inflammatory, anti-oxidative, endothelial protective properties, as well as hematology stabilization. These features led to the alleviation of lung injuries and neurologic disorders, and significant improvement of DCS prognosis.

Clinically, UTI has already been adopted in treating diseases such as acute pancreatitis, circulatory shock, and systemic inflammatory response syndrome conditions including burns and acute respiratory distress syndrome (ARDS) in China and Japan (Nakahama et al., 1999; Tsujimura et al., 2008; Wang et al., 2016). The results in the present study not only confirm the protective effects of UTI in treating DCS, especially for severe cases, but also prove UTI is powerful in coping with the inflammation cascade response and endothelium related injuries. These evidences provide additional support for replacing steroids with UTI, at least partially, in the treatment of critical diseases characterized by inflammatory responses. No obvious side effect has been observed in past observations, either clinically or experimentally. All these properties favor applying UTI in treating various critical diseases.

In conclusion, this is the first study to explore the potential effects of UTI on DCS using a rabbit model. A single intravenous injection of UTI immediately following decompression exerted significant protective effects against DCS in a dose-response manner, especially for severe cases. The beneficial effects of UTI on DCS were attributed to its anti-inflammatory, anti-oxidative, endothelial protective properties, as well as inhibition of cell adhesion. Depending on the clinical routine in using UTI, it is thought repeated administrations will likely gain further benefits. Regarding that steroids are no longer recommended for the treatment of DCS, UTI may be an ideal substitute. Further explorations in large animals and divers are encouraged.

## Data Availability Statement

The raw data supporting the conclusions of this manuscript will be made available by the authors, without undue reservation, to any qualified researcher.

## Ethics Statement

The animal study was reviewed and approved by the Ethics Committee for Animal Experiment of the Naval Medical University.

## Author Contributions

WX, WM, and LQ designed the experiments. WM, LQ, CL, and HY conducted the experiments. WM, LQ, and XZ contributed to data analyses and interpretation of the results. WX, WM, LQ, and CL wrote the manuscript and prepared all the figures and tables. KZ revised the manuscript.

## Conflict of Interest

The authors declare that the research was conducted in the absence of any commercial or financial relationships that could be construed as a potential conflict of interest.
